# Occupational health disparities among U.S. long-haul truck drivers: the influence of work organization and sleep on cardiovascular and metabolic disease risk

**DOI:** 10.1371/journal.pone.0207322

**Published:** 2018-11-15

**Authors:** Adam Hege, Michael K. Lemke, Yorghos Apostolopoulos, Sevil Sönmez

**Affiliations:** 1 Appalachian State University, Department of Health & Exercise Science, Boone, NC, United States of America; 2 University of Houston-Downtown, Department of Social Sciences, Houston, TX, United States of America; 3 Texas A&M University, Complexity & Computational Population Health Group, College Station, TX, United States of America; 4 Texas A&M University, Department of Health & Kinesiology, College Station, TX, United States of America; 5 University of Central Florida, College of Business Administration, Orlando, FL, United States of America; Universitat de Valencia, SPAIN

## Abstract

**Objective:**

The organization of work has undergone vast transformations over the past four decades in the United States and has had profound impacts on worker health and wellbeing. The profession of commercial truck driving is one of the best examples. Particularly for long-haul truck drivers, changes in work organization have led to disproportionately poor physiological, psychological, and sleep health outcomes.

**Methods:**

The present study examined disparities in cardiometabolic disease risk among long-haul truck drivers and the general population, and the influence of work organization and sleep in generating these outcomes. Researchers collected survey data from 260 drivers, and blood assay samples from 115 of those drivers, at a large highway truck stop in North Carolina. Comparisons were made for cardiovascular and metabolic risk against the 2011–2012 National Health and Nutrition Examination Survey (NHANES). In addition, logistic regression was used to explore predictive relationships between work organization and sleep and risk for cardiovascular and metabolic disease.

**Results:**

There were statistically significant mean differences between the long-haul truck driver sample and the NHANES sample for both cardiovascular (3.71 vs. 3.10; p <0.001) and metabolic (4.31 vs. 3.09; p <0.001) disease risk. The truck driver sample was less physically active and had lower HDL cholesterol along with greater levels of smoking, BMI, and metabolic syndrome diagnosis. More years of driving experience and poor sleep quality were statistically significant predictors for both cardiovascular and metabolic disease risk.

**Conclusions:**

Study findings implicate elements of the occupational milieu experienced by long-haul truck drivers that induce disproportionate cardiometabolic disease risk. Sleep quality, largely compromised by poor work conditions and workplace environments, plays a significant role in increased risks for cardiometabolic disease. There is an urgent need for longitudinal studies of this critical occupational sector as well as intervention research centered on policy and systems level change.

## Introduction

Work and employment conditions are fundamental to health, wellbeing, and quality of life [1–3]. The last forty years have been marked by drastic changes in work conditions and influenced by the forces of globalization and neoliberal policies, within the United States (U.S.) and globally [4–7]. Among these changes are declines in union membership, reduced power of collective bargaining, greater job insecurity, and increased work-related stressors. Furthermore, employees have less control over their work conditions than that of previous generations while they work longer and more unpredictable hours and receive fewer benefits and negligible wage increases from their employers. As a result, these unfortunate shifts have negatively impacted some populations more than others—in particular those identified as having lower socioeconomic status in society and greater vulnerability to health disparities [1, 3, 8, 9]. Also during these last forty years, using epidemiologic evidence, public health scholars have established causal linkages between shorter duration sleep [10], obesity [11], and increases in prevalence for both diabetes and cardiovascular disease (CVD) on a global scale [12]. Policymakers have effectively shaped the ‘organization of misery,’ which are in essence societal structures (including work conditions) that are responsible for health inequities around the world [13].

The study of work organization typically focuses on the impacts of upstream elements of policy and organizational decisions on downstream working conditions [4, 8, 9]. Numerous work organization-related factors have been shown to influence health at the level of individuals and subsequently produce health disparities across occupational sectors at the population level [4, 14, 15]. The direct psychosocial and physical exposures that workers experience daily (i.e., long work hours, shift work, lack of support from supervisors and coworkers) have been associated with poorer health behaviors [16, 17], obesity and cardiometabolic disease (CMD) [18–21], sleep problems [22–24], mental illness [25–27], as well as increased risks for accidents and injuries [28, 29]. Long work hours and shift work have received more research attention in relation to worker health than other work characteristics. There is growing evidence that work schedules play a significant role in diminishing sleep quality and duration, which can lead to metabolic disturbances that in turn create pathways for the development of metabolic diseases, such as obesity, Type 2 diabetes, or metabolic syndrome [30–34]. Similarly, job-related stress has implications for behavioral responses (e.g., poor dietary intake, weight gain, physical inactivity, poor sleep) that, when combined with physiological stress, are associated with increased risks for CVD [19; 35–39]. Beyond these physical health concerns, adverse work conditions have been associated with increased fatigue, decreased productivity while on the job, and a diminished work-life balance, resulting in poorer health behaviors, depression, and other mental illnesses [40–42].These connections between work organization and employee health have pushed the National Institute for Occupational Safety and Health (NIOSH) to make work organization a national priority for occupational health and safety researchers [1, 6, 43].

Specific industries most often associated with poor health outcomes due to detrimental work organization and conditions include agriculture, construction, healthcare, and transportation [4, 44, 45]. As members of the transportation sector, the nearly two million U.S. long-haul truck drivers (LHTD)—whose unique work organization requires spending extended periods of time away from home—have undergone major changes in their work organization over the last several decades [46]. These drivers are particularly prone to physiological and psychological stressors that include long work hours, frequent shift work, limited control over work conditions, excessive time pressures, burnout, work-home conflicts, social isolation, and chronic lack of (or access to) healthcare resources [47–51]. LHTDs are exposed to workplace environments that are unconducive to healthy behaviors [52–54]. Because of the unique combination of risk exposures, undesirable conditions, and chronic stress, long-haul trucking is considered one of the riskiest and unhealthiest occupations—characterized by excessive fatigue, sleep disorders, and accident and injury risks, and CMD risks [55–58]. It must be noted, however, that the degree of CMD risks for drivers resulting from their work conditions is unclear.

Given inconclusive evidence and shortcomings in the scientific literature regarding work organization and cardiometabolic risks, especially among long-haul truck driver populations, the current study has two primary objectives. First, to better understand the impacts of the unique work organization characteristics experienced by LHTDs, we sought to better explicate disparities in CMD risks compared to the general U.S. population. Second, to better understand the influence of the unique occupational milieu of long-haul truck driving on chronic disease risk, we explored predictive relationships between work organization and sleep factors and subsequent risks for CVD and CMD.

## Methods

### Study setting and sample

The Institutional Review Board at the University of North Carolina-Greensboro approved the study. Written consent was obtained from all participants involved in the study. Data collection for this study of U.S. LHTDs took place at a large truck stop located in central North Carolina. The study design and data collection methods have been described in greater detail in previous papers using this dataset [55–57]. In brief, a nonexperimental, cross-sectional, interviewer-administered survey (Truck Sleep Disorders Survey [TSLDS]) was used to collect data from 262 U.S. LHTDs. Due to missing data from two study participants, the final sample size for statistical interpretation was 260 drivers. Interviewer-administered surveys were completed between 6:00–11:00 p.m. with drivers who were screened and determined to have an overnight layover. These drivers were asked to return to the data collection location between 4:00–6:00 a.m. the next morning following a 10-hour fast to provide a blood serum sample. Serological samples were collected from a total of 115 drivers, from the antecubital space in either arm using an aseptic technique. Blood samples were stored in ice and transported to the university’s exercise physiology lab for analysis using ELISA systems and the EPOCH plate reader (BioTek, Winooski, VT). For the current study, researchers focused on variables related to the possible relationships between work environment and organization, perceived stress, sleep duration and quality, and cardiometabolic health measures.

### Study measures

#### Perceived stress, health behavior and medication use for high blood pressure

Researchers were interested in how drivers perceived their stress levels, as well as health behaviors commonly associated with CMD risk. For perceived stress, responses were grouped as none-to-mild or moderate-to-chronic stress. Researchers considered “perceived stress” to be a comprehensive indicator of the impact of the work conditions on the driver. Drivers were asked about their daily time spent exercising (with responses ranging from no time, less than one hour, to 1+ hours); whether they smoke tobacco; and whether they use prescribed medications for high blood pressure.

#### Work organization

Drivers were asked a series of questions about characteristics related to their job. Key characteristics included: years of driving experience; miles they drove each week; number of days on the road per month; number of daily work hours; irregularities within daily work schedules; pace of work; and experiences with work-related time pressures. Responses to questions on driving mileage and years of driving experience were recorded as continuous values, while the other work organization variables were organized categorically. With driving mileage, researchers were most concerned about the 2,500 mile per week cutoff (500 miles per day on average), and therefore, grouped as such. The 2,500 mile per week cutoff allowed for equitable variance across the sample; in addition, drivers most frequently report between 2,000 and 3,000 miles per week. For years of driving experience, researchers were most interested in the distinction between 10 years of experience and less compared with drivers on the road for more than 10 years (an indication of the accumulation of years on the road associated with work experiences). Much like with driving mileage, the 10-year threshold allowed for equitable variance across the sample. As for the numbers of consecutive days spent on the road, drivers were asked in a five day sequence as follows: less than 5 days, 6–10 days, 11–15 days, 16–20 days, 21–25 days, 26–30 days, over one month, and more than two months. In terms of days on the road, researchers considered 21 or more days on the road to signify that time on weekends or outside of a traditional five day workweek were being experienced among drivers. Regarding their work hours, drivers were asked in a one hour sequence; and with so few drivers having lower number of work hours, researchers grouped this variable as into 0 = <11 hours, 1 = >11 hours. This cutoff was used because of rules that permit drivers to be on the road up to 11 hours per day. [59] Drivers were asked about specific experiences with shift work indicators to include the irregularity of their daily schedules with response selections including “same each day” and “different each day.” Regarding work pressures, drivers were asked about the frequency of having a fast pace of work and time pressures, with possible responses including “never or rarely” or “sometimes, frequently, or always.”

#### Sleep

Sleep duration for both workdays and non-workdays were provided by drivers in terms of the number of hours of sleep they were able to get during a 24-hour period. National Sleep Foundation recommendations [60] show that adults should seek between seven and nine hours of sleep per day. Drivers were asked about their sleep quality, on both workdays and non-workdays simply by “how often do you feel that you get a good night’s sleep?” Respondents were given answer options of “never or rarely” and “almost every night or every night.” Sleep duration and sleep quality variables were reverse coded for further analysis. Specifically, sleep duration was coded as 0 = 7+ hours of sleep, 1 = <7 hours, while sleep quality was coded as 0 = good night’s sleep, 1 = poor night’s sleep. Drivers were also asked if they had been diagnosed with sleep apnea with “yes” or “no” responses available to them.

#### Cardiovascular and metabolic disease risks

Because the focus of this study is on CVD and metabolic disease risk among the LHTDs, researchers used the Framingham Global CVD Risk Score [61] and the National Institute of Diabetes and Digestive and Kidney Diseases (NIDDK) [62] guidelines to determine these risks respectively. The Framingham Global CVD Risk score provides a 10-year estimate of one’s risk for developing CVD [63], while the NIDDK guidelines focus on the risk for developing Type II diabetes or metabolic syndrome [64]. For CVD risk, researchers used the following seven characteristics: age (≥40); gender (male); total cholesterol (≥200 mg/DL); HDL cholesterol (≤40 mg/dL); smoker; systolic blood pressure (≥140 mm/Hg); and the use of blood pressure medication. For metabolic disease risk, researchers used the following 10 characteristics: (age ≥45); race (non-white); BMI (≥25); blood glucose (≥100 mg/dL); diastolic blood pressure (≥90 mm/Hg); systolic blood pressure (≥140 mm/Hg); HDL cholesterol (≤35 mg/dL); triglycerides (≥250 mg/dL); physically inactive; and meeting three or more metabolic syndrome diagnosis characteristics (waist circumference ≥102 cm; triglycerides ≥150 mg/dL; systolic blood pressure ≥130 mm/Hg; diastolic blood pressure ≥85 mm/Hg; total blood pressure ≥130/85 mm/Hg; and glucose ≥110 mg/dL). There are, of course, other risk factors, but we were able to only use these due to the available data.

#### National Health and Nutrition Examination Survey (NHANES)

In this study, researchers were interested in making comparisons with national data to examine for possible disparities that exist between our sample of long-haul truck and the general U.S. population. We made use of the 2011–2012 publicly available data set [65] and filtered the sample to meet our study characteristics (i.e., only males, 18 years of age). For this study, we computed the mean and standard deviations for the following characteristics of the NHANES sample (only males): age; race; physical activity level; smoking level; use of blood pressure medication; BMI; blood pressure; HDL cholesterol; blood glucose; triglycerides; and number of metabolic syndrome (MetS) characteristics.

### Statistical analyses

Our initial analyses focused on examining cardiometabolic risk disparities among LHTDs. We used the mean and standard deviation of the NHANES sample, and from this number, provided the breakdown of the number of drivers that would fit within two standard deviations above or below the mean of the NHANES sample. Using the aforementioned Framingham and NIDDKD risk characteristics, we then calculated the frequency of scores from zero to seven for CVD risk and zero to 10 for metabolic risk. Quartile findings from the risk scores were used as outcome variables for further analysis (logistic regression).

Next, we focused on exploring the relationships between work organization, sleep, and cardiometabolic risk. First, bivariate correlation analyses were conducted to assess relationships between work organization variables and sleep variables, which served as predictors for the CMD risk outcomes (quartiles). We used the correlation results, along with our understanding of the profession, to group variables together into composite predictor variables. Lastly, using the four grouped variables and years of driving experience and sleep apnea diagnosis as predictor variables, we conducted ordinal logistic regression models for the CVD risk and the metabolic disease risk outcome variables. All statistical analyses were performed using SPSS 23.0 [66].

### Results

Of the respondents, 62.5% characterized their stress as a moderate to chronic level and 70.4% of the drivers reported being paid by the number of miles driven. The mean number of years driving was nearly 15, with 56.2% of the sample having more than 10 years of driving experience. Drivers reported an average of just under 3,000 miles of driving per week, with 54.6% driving an average of over 2,500 miles per week; 84.6% of drivers were on the road 21 or more days per month. Regarding work schedules, 70% worked more than 11 hours per day, and more than four in five drivers (82.7%) experienced a work schedule that varies from day-to-day. Further, 68% of drivers reported a fast work pace and 77.7% reported experiencing time pressures. In response to questions on sleep, drivers reported longer sleep duration on non-workdays (average of 8.27 on non-workdays vs. 6.95 hours on workdays) as well as improved sleep quality (16.7% reporting “never or rarely getting a good night’s sleep on non-workdays vs. 38.1% reporting the same for workdays). Thirty drivers (11.5% of the sample) reported being diagnosed with sleep apnea.

As shown in Table 1, regarding CVD risk indicators, only 11.4% of drivers had cholesterol higher than 200 mg/dL, while 65.2% had HDL cholesterol less than 40 mg/dL. Nearly 40% were identified as smokers and 26.2% had systolic blood pressure of greater than 140 mm/Hg. For metabolic disease risks, 88.9% of drivers had a BMI of 25 or greater and 58% had three or more indicators for metabolic syndrome. In addition, 27% had blood glucose higher than 100 mg/dL and more than one in five drivers had blood pressure readings higher than the established criterion. Lastly, some drivers were found to have low levels of HDL cholesterol (53.0% had lower than 35 mg/dL) along with low levels of physical activity (39.4% were considered sedentary).

**Table 1:** Cardiovascular and metabolic disease risk of long-haul truck driver sample.

**Cardiovascular Disease Risk**			
	Mean (SD)	Criterion	n (%)–Meeting risk
*Age*		≥40	191 (73.5)
*Gender*		Male	260 (100.0)
Total Cholesterol*	168.16 (30.21)	≥200 mg/dL	13 (11.4)
HDL Cholesterol*	35.08 (10.67)	≤40 mg/dL	75 (65.2)
*Smoker*		Current smoker	103 (39.6)
*Systolic Blood Pressure*	128.87 (18.63)	≥140 mm/Hg	68 (26.2)
*Blood Pressure Medication*		Yes	65 (25.0)
**Metabolic Disease Risk**			
	Mean (SD)	Criterion	n (%)–Meeting risk
*Age*		≥ 45	158 (60.8)
*Race (31*.*6% AA*, *7*.*5% Hispanic*, *2*.*6% Other)*		Non-white	111 (42.7)
*BMI*	33.40 (7.22)	≥ 25	233 (88.9)
Blood Glucose*	92.11 (26.30)	≥ 100 mg/dL	31 (27.0)
*Diastolic Blood Pressure*	81.42 (11.25)	≥ 90 mm/Hg	54 (20.8)
*Systolic Blood Pressure*	128.87 (18.63)	≥ 140 mm/Hg	68 (26.2)
HDL Cholesterol*	35.08 (10.67)	≤ 35 mg/dL	61 (53.0)
Triglycerides*	164.12 (91.80)	≥ 250 mg/dL	12 (10.4)
*Physical Activity Level*		Inactive/Sedentary	102 (39.4)
3 or more Metabolic Syndrome characteristics*	2.73 (1.25)	3 or more characteristics	66 (58.0)

*Only includes drivers in which a blood sample was collected (n = 115).

When comparing the long-haul truck driver sample with the NHANES data, we found statistically significant differences in the means in terms of the number of risk factors. More specifically, for CVD risk factors (3.71 vs. 3.10; p <0.001) and metabolic disease risk factors (4.31 vs. 3.09; p <0.001), the truck driver sample exhibited a greater mean number of risks. In looking at specific risks (illustrated in Table 2), smoking and sedentariness were found to be much more prevalent among the driver sample. Specifically, 42.3% of the truck driver sample was at least one standard deviation greater than the average of smoking among the NHANES sample. In addition, findings for BMI and blood pressure were much higher among the driver sample; in fact, 43.9% of the truck driver sample had a BMI of at least more than one standard deviation from the mean of the NHANES sample and nearly 30% of truckers did so for systolic blood pressure and nearly 50% for diastolic blood pressure. With these findings, it was not surprising that the drivers also had much lower HDL cholesterol levels (62.8% at least one standard deviation less than the mean) and met more characteristics for metabolic syndrome diagnosis (67.3% at least one standard deviation more than the mean).

**Table 2:** Distribution of cardiometabolic risk factors among LHTDs compared to NHANES (2011–2012)* sample.

	SD > - 2 n (%)	- 2 ≥ SD ≤ - 1 n (%)	- 1 ≥ SD ≤ 0 n (%)	0 ≥ SD ≤ 1 n (%)	1 ≥ SD ≤ 2 n (%)	SD > 2 n (%)
*Age*	—	13 (5.0)	113 (43.5)	127 (48.8)	7 (2.7)	—
*Race*	—	—	149 (57.3)	111 (42.7)	—	—
*Physical Activity Level*	158 (60.8)	—	102 (39.2)	—	—	—
*Smoking*	—	113 (43.5)	31 (11.9)	6 (2.3)	44 (16.9)	66 (25.4)
*Blood Pressure Medication*	65 (24.9)	—	—	196 (75.1)	—	—
*BMI*	—	—	33 (12.7)	113 (43.5)	81 (31.2)	33 (12.7)
*SBP*	1 (0.4)	5 (1.9)	64 (24.6)	112 (43.1)	60 (23.1)	18 (6.9)
*DBP*	—	2 (0.8)	11 (4.2)	120 (46.2)	102 (39.2)	25 (9.6)
HDL-C**	14 (12.4)	57 (50.4)	34 (30.1)	8 (7.1)	—	—
Blood Glucose**	—	27 (23.5)	65 (56.5)	14 (12.2)	7 (6.1)	2 (1.7)
TG**	—	—	43 (37.4)	51 (44.3)	15 (13.0)	6 (5.2)
MetS Characteristics**	—	5 (4.4)	10 (8.8)	22 (19.5)	34 (30.1)	42 (37.2)

*Filtered NHANES dataset to match the truck driver sample demographic characteristics.

**Only includes drivers from whom blood samples were obtained.

When assessing correlations between work organization and sleep variables, statistically significant relationships were found (Table 3). In particular, perceived stress was correlated with a higher frequency of a fast work pace and time pressures. A positive relationship was found between sleep duration and for sleep quality on both work and non-workdays. The years of driving experience as well as sleep apnea diagnosis—neither of which are directly work organization variables exhibited very limited correlations with any of the other variables. The remaining four variables (miles driven, days on road, work hours, schedule irregularity) were not statistically correlated, but based on our understanding of the profession, made logical sense to group together to represent work organization. Therefore, the six predictor variables for logistic regression analyses became: years of driving experience; stress (perceived stress, fast work pace, time pressures); sleep duration (workday and non-workday); sleep quality (workday and non-workday); sleep apnea diagnosis; and work organization (driving miles, days on road, work hours, irregularity of schedule).

**Table 3:** Correlation between work organization and sleep variables among LHTDs.

	Perceived Stress	Years Driving	Miles Driven	Days on the Road	Work Hours	Work Schedule	Fast Pace	Time Pressures	Sleep Duration (workday)	Sleep Duration (non-workday	Sleep Quality (workday)	Sleep Quality (non-workday)	Sleep Apnea
*Perceived Stress*	—	-0.02	0.03	0.04	0.04	0.04	0.24**	0.22**	0.13*	0.02	0.16*	0.16*	0.06
*Years of Driving*	-0.02	—	0.04	-0.07	0.01	0.07	-0.08	-0.06	-0.01	0.05	-0.07	-0.02	-0.07
*Miles per week*	0.03	0.04	—	0.04	0.07	-0.01	-0.01	0.09	0.01	0.04	0.11	0.06	0.06
*Days on road per month*	0.04	-0.07	0.04	—	0.07	0.06	0.04	0.10	-0.03	-0.08	-0.02	0.02	0.02
*Work hours per day*	0.04	0.01	0.07	0.07	—	0.06	0.10	0.10	0.06	0.04	0.06	0.07	0.04
*Irregularity of daily schedule*	0.04	0.07	-0.01	0.06	0.06	—	0.14*	0.00	0.06	0.10	0.06	0.04	0.03
*Fast pace of work*	0.24**	-0.08	-0.01	0.04	0.10	0.14*	—	0.45**	0.12*	0.01	0.15*	0.14*	0.04
*Time pressures*	0.22**	-0.06	0.09	0.10	0.10	0.00	0.45**	—	0.09	-0.07	0.13*	0.09	0.04
*Sleep duration (workday)*	0.13*	-0.01	0.01	-0.03	0.06	0.06	0.12*	0.09	—	0.39**	0.31**	0.15*	-0.06
*Sleep duration (non-workday)*	0.02	0.05	0.04	-0.08	0.04	0.10	0.01	-0.07	0.39**	—	0.16*	0.25**	0.12
*Sleep quality (workday)*	0.16*	-0.07	0.11	-0.02	0.06	0.06	0.15*	0.13*	0.31**	0.16*	—	0.50**	0.08
*Sleep quality (non-workday)*	0.16*	-0.02	0.06	0.02	0.07	0.04	0.14*	0.09	0.15*	0.25**	0.50**	—	0.15*
*Sleep apnea diagnosis*	0.06	-0.07	0.06	0.02	0.04	0.03	0.04	0.04	-0.06	0.12	0.08	0.15*	—

** p < 0.01;

* p < 0.05.

In the regression model on CVD risk, the model was statistically significant (*X*^*2*^ *= 25*.*77*, *p < 0*.*001)* and a driving experience of 10 years or less resulted in 81% reduced odds (OR = 0.19) and good sleep quality resulted in 59% reduced odds (OR = 0.41) of an increasing CVD risk. For the metabolic disease risk model, which was statistically significant (*X*^*2*^ *= 15*.*25*, *p < 0*.*01)*, a driving experience of 10 years or less resulted in 63% reduced odds (OR = 0.37) and good sleep quality resulted in 62% reduced odds (OR = 0.38) of an increasing CVD risk. Table 4 provides the findings from the logistic regression models.

**Table 4:** Logistic regression models for CMD risk. The analyses only includes LHTDs from whom blood samples were obtained to test for cardiometabolic risks.

	Wald *X*^*2*^	Odds Ratio	95% CI	*p*
**Cardiovascular Risk**				
Driving Experience (10 years or less) Overall Stress (reduced overall stress) Sleep Duration (good sleep duration) Sleep Quality (good sleep quality) Sleep Apnea Diagnosis (no diagnosis) Work Organization (improved scheduling) *X*^*2*^ *= 25*.*77*, *p < 0*.*001* *Cox and Snell R*^*2*^ *= 0*.*23* *Nagelkerke R*^*2*^ *= 0*.*24*	17.04 0.39 0.06 4.24 2.24 0.19	0.19 1.28 1.10 0.41 0.44 1.18	0.09, 0.42 0.60, 2.70 0.52, 2.34 0.18, 0.96 0.15, 1.29 0.56, 2.45	0.01*** 0.53 0.81 0.04* 0.14 0.67
**Metabolic Risk**				
Driving Experience (10 years or less) Overall Stress (reduced stress) Sleep Duration (good sleep duration) Sleep Quality (good sleep quality) Sleep Apnea Diagnosis (no diagnosis) Work Organization (improved scheduling) *X*^*2*^ *= 15*.*25*, *p < 0*.*01* *Cox and Snell R*^*2*^ *= 0*.*14* *Nagelkerke R*^*2*^ *= 0*.*15*	6.81 0.44 1.23 5.19 0.27 0.03	0.37 1.29 0.65 0.38 0.76 1.07	0.18, 0.78 0.61, 2.72 0.31, 1.39 0.16, 0.87 0.26, 2.17 0.52, 2.22	0.01** 0.51 0.27 0.02* 0.60 0.86

*** p < 0.001;

** p < 0.01;

* p < 0.05.

### Discussion

Overall, our findings suggest that, cumulatively, the unique and layered risks that LHTDs are exposed to that are endemic to their profession play important roles in generating CMD risk disparities compared to the general U.S. male population. These excessive risks constitute underappreciated and underestimated economic and public health risks, as they pose threats to the drivers themselves [67, 68], jeopardize U.S. trucking companies [69, 70], place strain on the U.S. healthcare system [71, 72], and threaten the lives of other roadway users in the event of highway accidents [72, 73]. Thus, broad and immediate preventive action is warranted to address these disparities.

### Long-haul truck drivers’ cardiometabolic disease risk

Findings from this study indicate that the occupational milieu of long-haul trucking generates excessive cardiometabolic risks. Previous studies have similarly identified this profession as posing numerous risks to cardiometabolic health [74], both through the impacts of key work organization forces (e.g., scheduling, job stress) [75] and the impacts of work-related physical and psychosocial strains on CMD-related behavioral risks [76]. As a result, CMD risks appear to cluster among LHTD compared to other working populations [77]. Such clustering of risks may shorten LHTD life expectancies, which are believed to be significantly reduced compared to other populations [67]. Overall, the findings here highlight how CMD risks indeed cluster among LHTD compared to the general U.S. male population, resulting in significantly higher CVD and CMD risks that contribute to heightened prevalence of various CMD diseases, and at earlier ages [69, 73, 77–84].

The current study identifies specific CMD risks; in particular, low levels of physical activity, frequent smoking, high levels of obesity, low levels of HDL cholesterol, and heightened MetS prevalence, which appears to be especially the case among LHTDs. These results corroborate findings of prior studies that these risks are common among LHTDs. For example, LHTDs have frequently been found to have low levels of physical activity, with relatively few drivers meeting recommended physical activity guidelines or regularly engaging in physical activity of any type [67, 73, 77, 85, 86]. Similarly, other studies have regularly shown more heightened levels of smoking [77, 79, 85, 86] and obesity [77, 79, 81, 85–88] among LHTDs compared to other populations. Disparities regarding HDL cholesterol and MetS prevalence are less established in the existing literature, although extant studies suggest that dyslipidemia is problematic among LHTDs [77, 89], that MetS is more common among transportation workers as a whole [90], and that frequently-used MetS diagnostic criteria are frequently clustered among LHTDs [77, 91]. These specific CMD risks have been independently associated with CMD pathogenesis, both in the general population and specifically among LHTDs [68, 70, 71, 88, 92–97].

### The impacts of experience, sleep, and work organization on Cardiometabolic disease risk

Increases in driving experience were associated with heightened CMD risks, suggesting a dose-response relationship between tenure as a LHTD and chronic disease outcomes. Prior studies of working populations have found that work-related stress has a similar influence on CVD risk, especially for developing coronary heart disease among employees aged 50 and younger [98]. Stress, however, was not a significant predictor in either model and this relationship remains unclear. Studies investigating LHTDs in particular have found relationships between increasing age and hypertension, although not experience per se [73], while other LHTD studies have found that younger drivers are most at-risk for health complications [87]. The connection between work experience and chronic disease risk among LHTDs warrants further attention to clarify the somewhat contradictory conclusions in the scientific literature.

Sleep quality was also a significant predictor in both the CVD and metabolic risk regression models. Sleep quality has been found to be poor among LHTD and has been attributed to numerous work organization and health behavior risks, including poor physical activity, smoking, long work hours, frequent shift work, and low SES [87, 99–105]. Poor sleep quality has been established as a risk factor for poor self-perceived health, heightened job stress, poor job satisfaction, psychological distress, poor dietary choices, MetS diagnosis, dyslipidemia, and roadway accident risk among LHTDs as well [91, 100, 101, 106–109], although this study represents the first time that it has been tied to global CVD and metabolic risk. Other studies have found poor sleep quality associated with less driving experience [106, 110], which suggests that adaptive mechanisms or strategies that are learned by more experienced drivers to cope with the psychological and physical strains of long-haul trucking are harmful for cardiometabolic health in the long-term.

While the connections between sleep and CMD risk are well established [93, 111], in our analyses sleep *duration—*the length that drivers slept each night, was not a significant predictor. This finding was surprising, given the established connections between sleep duration and cardiometabolic risks among other populations [93, 111–113]. While it would be inappropriate to wholly disregard the importance of sleep duration among LHTDs, our findings suggest that the occupational milieu of long-haul trucking, where the aforementioned risks regularly compromise sleep quality, may powerfully mediate this relationship. Together, this points to the fallacy of placing sole emphasis on sleep duration, on behalf of both the trucking industry and federal policymaking bodies, and suggests that newfound efforts should target LHTD sleep quality to protect drivers from CMD risk, which echoes similar calls from other researchers vis-à-vis sleep quality and roadway safety [108, 110].

For trucking industry and government stakeholders, numerous potential pathways exist to improve LHTD sleep quality, and many of these pathways have other direct influences on cardiometabolic health as well. Innovative workplace health and wellness programs can be implemented to improve sleep quality, such as educating drivers about strategies to enhance sleep health, such as regular exercise and avoiding consumption of caffeine or other stimulants before bedtime; providing resources or incentives to help drivers to manage their weight, in order to reduce risk for sleep disorders (i.e., sleep apnea) that deteriorate sleep quality; and implementing sleep disorder screening and treatment programs [71, 114–118]. However, given the risk-laden occupational environment of long-haul trucking, it is unlikely that workplace health and wellness programs alone will suffice in curbubg poor sleep quality and commensurate cardiometabolic risks. Thus, built environment improvements to worksites that are notorious for being detrimental to sleep and cardiometabolic health, [119] should also be addressed. Environmental modifications such as quieter sleeper berths, adequate in-cab temperature controls, improved safety and security, and improved ambient air quality can help drivers achieve better quality sleep [72, 120, 121].

Finally, existing policies should be modified, and new policies enacted, to improve work conditions to enhance LHTD sleep quality and to reduce cardiometabolic risks [85, 122]. At the trucking industry level, companies should make a concerted effort to improve driver scheduling to help ensure that rest periods coincide with circadian nadirs, which will require scheduling practices that actively avoid shift work [123]. Shippers and consignees must also be open to scheduling appointments during times that allow for such scheduling practices. Additionally, truckstop companies must modify their nutritional offerings and provide opportunities for physical activity (e.g., gyms, walking paths) to better support healthful behaviors among drivers [85, 124]. Federal policies should be modified to better support LHTD sleep quality and reduce cardiometabolic risk [125]. As mentioned earlier, new emphases should be placed on sleep quality, which will require reconsideration of Federal Motor Carrier Safety Administration (FMCSA) hours-of-service regulations that currently only pertain to sleep duration [126]. Action should also be undertaken at the federal level to screen, diagnose, and treat LHTDs with sleep apnea and other sleep disorders. A planned program to make sleep apnea testing mandatory among LHTDs was recently reversed by the Trump administration; however, federal involvement will likely be instrumental in improving sleep quality among truck drivers at the population level and improving cardiometabolic outcomes [127]. Finally, consideration should be given to research which untangles the complicated relationships between LHTD occupational milieu, fatigue, and sleep health, which could then inform policies that address other correlates with sleep quality. For example, research in other populations has shown that work-related fatigue is associated with numerous factors, such as income, depression, social support, home life, work hours, and work-related strain and stressors [40, 41, 128]. Critical in these efforts will be developing consensus regarding how sleep quality and fatigue are conceptualized and measured among researchers, as these conceptual and methodological problems continue to plague related LHTD research endeavors [129]. Understanding how sleep quality, sleep duration, and fatigue are influenced by the unique occupational milieu of LHTD may lead to effective federal policies that improve sleep health and lead to improved CMD and roadway safety outcomes in this vulnerable population.

The current study has four primary limitations. First, the overall sample size is relatively small, with 262 LHTDs and serological samples collected from only 115. This diminished our statistical power, which may have led to important factors failing to meet the criterion for statistical significance in our regression models. However, the similarities of our findings with other relevant studies indicate that our sample is representative. Second, the potential for selection bias is an important limitation to consider when interpreting our results. Drivers may have refused to participate in our study, and especially the blood draw portion, for a myriad of reasons. Third, our data do not include all the NIDDK measures for metabolic risk, such as family history, which may have impacted the accuracy of our analyses and conclusions. Finally, our sample includes only male drivers, which is one of the CVD and metabolic disease risk factors; as a result, there was no variance for this risk factor in our sample.

## Conclusions

In summary, our study supports previous research showing that U.S. LHTDs encounter occupation-related cardiovascular and metabolic health disparities. Specifically, the findings showed that poor sleep quality and increased exposure to the occupational setting of commercial driving (i.e., more work experience) are associated with CMD risk. With years of experience being a significant predictor of disease risk, there is an urgent need for longitudinal studies of this critical occupational sector. Additionally, intervention research should focus on improving driver sleep quality and other detrimental elements of the LHTD work environment, and especially policy and systems level change, rather than individual-based behavioral change.

## Supporting information

S1 Dataset Truck driver sleep study data. This is the fully anonymized data set used for this study.
(XLSX)

S1 Codebook Truck driver sleep study codebook. This is the codebook that was used when tabulating the data.
(DOCX)
