# Supplementary material for: Occupational health disparities among U.S. long-haul truck drivers: the influence of work organization and sleep on cardiovascular and metabolic disease risk
Source: PLoS One. 2018 Nov 15;13(11):e0207322. doi: 10.1371/journal.pone.0207322 (PMC6237367; doi:10.1371/journal.pone.0207322)
Supplement: S1 Codebook — This is the codebook that was used when tabulating the data. (DOCX) [file pone.0207322.s002.docx]

**Appendix A. *Trucker Sleep Disorders Survey (TSLDS)***

**LONG-HAUL TRUCKER DRIVERS SLEEP SURVEY**

**CODE BOOK**

| **PART** | | **VARIABLE** | | | | | | | **CODE** | **SPACES** |
| --- | --- | --- | --- | --- | --- | --- | --- | --- | --- | --- |
| PID | | Participant ID number | | | | | | | 001 through 262 | 3 |
| INT | | Research team interviewer | | | | | | |  | 2 |
| DATE | | Date of interview | | | | | | |  | 8 |
| TIME | | Time of interview | | | | | | |  | 7 |
| **BIOMETRIC DATA** | | | | | | | | | | |
| **NAME** | | | **CALCULATE** | | | | **VARIABLE** | | **CODE** | **SPACES** |
| Pulse | | | Mean of two pulse rate readings | | | | Pulse | | Ex: 75 | 3 |
| Systolic PB | | | Mean of two PB readings for systolic | | | | Systolic | | Ex: 120 | 3 |
| Diastolic PB | | | Mean of two PB readings for diastolic | | | | Diastolic | | Ex: 100 | 3 |
| Height | | | Calculate height into inches (5’5” = 65) | | | | Height | | Ex: 170 | 3 |
| Weight | | | Report weight as written | | | | Weight | | Ex: 360 | 3 |
| BMI | | | Weight/Height in inches x Height in inches x 703 | | | | BMI | | Ex: 30 | 2 |
| Sagittal | | | Mean of 3 measures of sagittal diameter (convert to inches) | | | | Sagital | | Ex: 120 | 3 |
| CircW | | | Mean of 3 measures of waist circumference (convert to inches) | | | | CirW | | Ex: 100 | 3 |
| Waist-Hip Ratio | | | Ratio of Waist:Hip | | | | WHpRatio | | Ex: |  |
| Waist-Height Ratio | | | Ratio of Waist:Height | | | | WHtRatio | | Ex: |  |
| CircH | | | Mean of 3 measures of hip circumference (convert to inches) | | | | CirH | | Ex:100 | 3 |
| CircUA | | | Mean of 3 measures of upper arm circumference (convert to inches) | | | | CirUA | | Ex: 40 | 2 |
| **BIOLOGICAL ASSAYS** | | | | | | | | | | |
| LipidCh-HDL | | |  | | | |  | |  |  |
| LipidCh-LDL | | |  | | | |  | |  |  |
| Glucose | | |  | | | |  | |  |  |
| Insulin | | |  | | | |  | |  |  |
|  | | |  | | | |  | |  |  |
| **SURVEY QUESTIONS** | | | | | | | | | | |
| **PART** | **QUESTN** | | | **VARIABLE** | **VARIABLE NAME** | **CODE** | | **VALUE** | | **SPACES** |
| **PART I – DRIVER TYPE** | | | | | | | | | | |
| Part 1 | 1 | | | P1Q1 | Driver type | 01  02  03  04 | | Company driver  For-hire  OO-Own authority  OO-Lease | | 2 |
|  |  |  |  |  |  | 05 | | String variable (write out) | | 10 |
| Part 1 | 2 | | | P1Q2 | Load type | 01  02  03  04  05 | | Full TL  LTL  Refrigerated  Specialized-bulk  Specialized-other | | 2 |
| Part 1 | 3 | | | P1Q3 | Years of driving | Years | | Write years reported | | 2 |
| Part 1 | 4 | | | P1Q4 | Miles per week (if reported in year, convert to week | Years | | Write miles reported | | 4 |
| Part 1 | 5 | | | P1Q5 | Days on road | 01  02  03  04  05  06  07  08 | | Less than 5  6-10 days  11-15 days  16-20 days  21-25 days  26-30 days  Over 1 month  More than 2 months | | 2 |
| Part 1 | 6 | | | P1Q6 | Hours work in a day | 01  02  03  04  05  06  07  08  09  10 | | Less than 6  6-7 hours  7-8 hours  8-9 hours  9-10 hours  10-11 hours  11-12 hours  12-13 hours  13-14 hours  Over 14 hours | | 2 |
| Part 1 | 7 | | | P1Q7 | Schedule | 00  01 | | Different every day  Same every day | | 2 |
| Part 1 | 8 | | | P1Q8 | Daily hours | 00  01 | | Different every day  Same every day | | 2 |
| Part 1 | 9 | | | P1Q9 | Days of week | 00  01 | | Different each week  Same each week | | 2 |
| Part 1 | 10 | | | P1Q10 | Work start | 01  02  97 | | Actual time A.M. *(round off to nearest full hour)*  Actual time P.M.  Varies | | 2 |
| Part 1 | 11 | | | P1Q11 | Work finish | 01  02  98 | | Actual time A.M.  Actual time P.M.  Varies | | 2 |
| Part 1 | 12 | | | P1Q12 | Hour awake: work days | 01  02  03  97  99 | | Actual time A.M.  Actual time P.M.  Range *(round off to nearest full hour)*  Varies  Don’t know/remember | | 2 |
| Part 1 | 13 | | | P1Q13 | Hour awake: nonwork days | 01  02  03  97  98  99 | | Actual time A.M.  Actual time P.M.  Range *(round off to nearest full hour)*  Varies  Does not apply  Don’t know/remember | | 2 |
| Part 1 | 14 | | | P1Q14 | Hour asleep: work days | 01  02  03  97  99 | | Actual time A.M.  Actual time P.M.  Range *(round off to nearest full hour)*  Varies  Don’t know/remember | | 2 |
| Part 1 | 15 | | | P1Q15 | Hour asleep: nonwork days | 01  02  03  97  98  99 | | Actual time A.M.  Actual time P.M.  Range *(round off to nearest full hour)*  Varies  Does not apply  Don’t know/remember | | 2 |
| Part 1 | 16 | | | P1Q16 | Fast pace of work | 00  01  02  03  04 | | Never  Rarely  Sometimes  Oftentimes  Always | | 2 |
| Part 1 | 17 | | | P1Q17 | Work under time pressure | 00  01  02  03  04 | | Never  Rarely  Sometimes  Oftentimes  Always | | 2 |
| Part 1 | 18 | | | P1Q18 | Repetitive work | 00  01  02  03  04 | | Never  Rarely  Sometimes  Oftentimes  Always | | 2 |
| Part 1 | 19 | | | P1Q19 | Opportunities to learn new things | 00  01  02  03  04 | | Never  Rarely  Sometimes  Oftentimes  Always | | 2 |
| Part 1 | 20 | | | P1Q20 | Support of co-workers | 00  01  02  03  04 | | Never  Rarely  Sometimes  Oftentimes  Always | | 2 |
| Part 1 | 21 | | | P1Q21 | Support of supervisor | 00  01  02  03  04 | | Never  Rarely  Sometimes  Oftentimes  Always | | 2 |
| **PART II – GENERAL HEALTH** | | | | | | | | | | |
| Part 2 | 22 | | | P2Q22 | Overall health | 01  02  03  04  05  06 | | Very Poor  Poor  Fair  Good  Very Good  Excellent | | 2 |
| Part 2 | 23 | | | P2Q23 | Stress level | 00  01  02  03  04  05 | | No stress  Mild stress  Moderate stress  High stress  Extreme stress  Acute/chronic stress | | 2 |
| Part 2 | 24 | | | P2Q24 | Alcohol consumption: workday | 00  01  02  03  04  99 | | None  1 drink  2-3 drinks  4-5 drinks  6+ drinks  Don’t know/remember | | 2 |
| Part 2 | 25 | | | P2Q25 | Alcohol consumption: nonwork day | 00  01  02  03  04  98  99 | | None  1 drink  2-3 drinks  4-5 drinks  6+ drinks  Does not apply  Don’t know/remember | | 2 |
| Part 2 | 26 | | | P2Q26a | Diagnosis: arthritis/rheumatism | 01  02  99 | | Yes  No  Don’t know/remember | | 2 |
| Part 2 | 26 | | | P2Q26b | Diagnosis: chronic back/neck pain | 01  02  99 | | Yes  No  Don’t know/remember | | 2 |
| Part 2 | 26 | | | P2Q26c | Diagnosis: high blood pressure/hypertension | 01  02  99 | | Yes  No  Don’t know/remember | | 2 |
| Part 2 | 26 | | | P2Q26d | Diagnosis: cardiovascular problems | 01  02  99 | | Yes  No  Don’t know/remember | | 2 |
| Part 2 | 26 | | | P2Q26e | Diagnosis: high cholesterol | 01  02  99 | | Yes  No  Don’t know/remember | | 2 |
| Part 2 | 26 | | | P2Q26f | Diagnosis: diabetes | 01  02  99 | | Yes  No  Don’t know/remember | | 2 |
| Part 2 | 26 | | | P2Q26g | Diagnosis: ulcer in stomach or intestine | 01  02  99 | | Yes  No  Don’t know/remember | | 2 |
| Part 2 | 26 | | | P2Q26h | Diagnosis: irritable bowel syndrome | 01  02  99 | | Yes  No  Don’t know/remember | | 2 |
| Part 2 | 26 | | | P2Q26i | Diagnosis: chronic heartburt | 01  02  99 | | Yes  No  Don’t know/remember | | 2 |
| Part 2 | 26 | | | P2Q26j | Diagnosis: asthma | 01  02  99 | | Yes  No  Don’t know/remember | | 2 |
| Part 2 | 26 | | | P2Q26k | Diagnosis: bronchitis or emphysema | 01  02  99 | | Yes  No  Don’t know/remember | | 2 |
| Part 2 | 26 | | | P2Q26l | Diagnosis: obstructive pulmonary disease | 01  02  99 | | Yes  No  Don’t know/remember | | 2 |
| Part 2 | 26 | | | P2Q26m | Diagnosis: lung cancer | 01  02  99 | | Yes  No  Don’t know/remember | | 2 |
| Part 2 | 26 | | | P2Q26n | Diagnosis: urinary or bladder problems | 01  02  99 | | Yes  No  Don’t know/remember | | 2 |
| Part 2 | 26 | | | P2Q26o | Diagnosis: bladder cancer | 01  02  99 | | Yes  No  Don’t know/remember | | 2 |
| Part 2 | 26 | | | P2Q26p | Diagnosis: chronic fatigue or low energy | 01  02  99 | | Yes  No  Don’t know/remember | | 2 |
| Part 2 | 26 | | | P2Q26q | Diagnosis: anxiety disorder | 01  02  99 | | Yes  No  Don’t know/remember | | 2 |
| Part 2 | 26 | | | P2Q26r | Diagnosis: depression | 01  02  99 | | Yes  No  Don’t know/remember | | 2 |
| Part 2 | 26 | | | P2Q26s | Diagnosis: frequent/severe headaches | 01  02  99 | | Yes  No  Don’t know/remember | | 2 |
| Part 2 | 26 | | | P2Q26t | Diagnosis: other (WRITE IN) | 01  02  99 | | Yes  No  Don’t know/remember | | 2 |
| Part 2 | 26 | | | P2Q26u | Diagnosis: other (WRITE IN) | 01  02  99 | | Yes  No  Don’t know/remember | | 2 |
| Part 2 | 26 | | | P2Q26v | Diagnosis: other (WRITE IN) | 01  02  99 | | Yes  No  Don’t know/remember | | 2 |
| Part 2 | 27 | | | P2Q27 | Medications prescribed | 01  02  03  04  05  06  07  08  09  10  11  12  13  14  15  16  17  18  19  20  21  22 | | Arthritis (WRITE IN MED)  Back pain (WRITE IN MED)  BP (WRITE IN MED)  CV problems (WRITE IN MED)  Cholesterol (WRITE IN MED)  Diabetes (WRITE IN MED)  Ulcer (WRITE IN MED)  IBS (WRITE IN MED)  Heartburn (WRITE IN MED)  Asthma (WRITE IN MED)  Bronchitis (WRITE IN MED)  Pulmonary (WRITE IN MED)  Lung cancer (WRITE IN MED)  Urinary (WRITE IN MED)  Bldr cancer (WRITE IN MED)  Fatigue (WRITE IN MED)  Anxiety (WRITE IN MED)  Depression (WRITE IN MED)  Headaches (WRITE IN MED)  Other 1 (WRITE IN MED)  Other 2 (WRITE IN MED)  Other 3 (WRITE IN MED) | | 10 |
| **PART III – GENERAL SLEEP** | | | | | | | | | | |
| Part 3 | 28 | | | P3Q28 | Work night hours of sleep | Hours  99 | | Write hours reported  Don’t know/remember | | 2 |
| Part 3 | 29 | | | P3Q29 | Nonwork night hours of sleep | Hours  98  99 | | Write hours reported  Not applicable  Don’t know/remember | | 2 |
| Part 3 | 30 | | | P3Q30 | Workday naps | Number  Number  99 | | Actual number of naps  Actual times dozed off  Don’t know/remember | | 2 |
| Part 3 | 31 | | | P3Q31 | Workday nap length | Minutes  Hours  99 | | Number of minutes  Number of hours  Don’t know/remember | | 2 |
| Part 3 | 32 | | | P3Q32 | Naps while working/not on road | Number  99 | | Number of times  Don’t know/remember | | 2 |
| Part 3 | 33 | | | P3Q33 | Nonwork day naps | Number  98  99 | | Actual number of naps  Does not apply  Don’t know/remember | | 2 |
| Part 3 | 34 | | | P3Q34 | Nonwork day nap length | Minutes  Hours  98  99 | | Number of minutes  Number of hours  Does not apply  Don’t know/remember | | 2 |
| Part 3 | 35 | | | P3Q35 | Falling asleep while working | 00  01  99 | | No  Yes  Don’t know/remember | | 2 |
| Part 3 | 36 | | | P3Q36 | Told dispatcher too tired to drive | 00  01  99 | | Never told  Never told for fear of firing  Don’t remember | | 2 |
|  |  |  |  |  |  | 02 | | String variable (write out) | | 10 |
| Part 3 | 37 | | | P3Q37 | Work nights: good night’s sleep | 00  01  02  03  99 | | Never  Rarely  Almost every night  Every night  Don’t know/remember | | 2 |
| Part 3 | 38 | | | P3Q38 | Nonwork nights: good night’s sleep | 00  01  02  03  99 | | Never  Rarely  Almost every night  Every night  Don’t know/remember | | 2 |
| Part 3 | 39 | | | P3Q39 | Caffeine consumption | Number  99 | | Calculate total number of ounces  Don’t’ know/remember | | 3 |
| Part 3 | 40 | | | P3Q40 | Tobacco consumption | Number  98  99 | | Number of packs/day  Does not apply  Don’t know/remember | | 2 |
| Part 3 | 41 | | | P3Q41 | Other substances | Number  98  99 | | Number of times/week  Does not apply  Don’t know/remember | | 2 |
|  |  |  |  |  |  | 97 | | Participant wanted to skip question | | 2 |
| Part 3 | 42 | | | P3Q42a | Doze off: sitting/reading | 00  01  02  03  04  99 | | Never  Less than once/week  1-2 times/week  3-4 times/week  5+ times/week  Don’t know/remember | | 2 |
| Part 3 | 42 | | | P3Q42b | Doze off: watching tv | 00  01  02  03  04  99 | | Never  Less than once/week  1-2 times/week  3-4 times/week  5+ times/week  Don’t know/remember | | 2 |
| Part 3 | 42 | | | P3Q42c | Doze off: sitting in public space | 00  01  02  03  04  99 | | Never  Less than once/week  1-2 times/week  3-4 times/week  5+ times/week  Don’t know/remember | | 2 |
| Part 3 | 42 | | | P3Q42d | Doze off: sitting/talking | 00  01  02  03  04  99 | | Never  Less than once/week  1-2 times/week  3-4 times/week  5+ times/week  Don’t know/remember | | 2 |
| Part 3 | 42 | | | P3Q42e | Doze off: after lunch | 00  01  02  03  04  99 | | Never  Less than once/week  1-2 times/week  3-4 times/week  5+ times/week  Don’t know/remember | | 2 |

| Part 3 | 42 | P3Q42f | Doze off: lying down to rest | 00  01  02  03  04  99 | Never  Less than once/week  1-2 times/week  3-4 times/week  5+ times/week  Don’t know/remember | 2 |
| --- | --- | --- | --- | --- | --- | --- |
| Part 3 | 42 | P3Q42g | Doze off: | 00  01  02  03  04  99 | Never  Less than once/week  1-2 times/week  3-4 times/week  5+ times/week  Don’t know/remember | 2 |
| Part 3 | 42 | P3Q42h | Doze off: | 00  01  02  03  04  99 | Never  Less than once/week  1-2 times/week  3-4 times/week  5+ times/week  Don’t know/remember | 2 |
| Part 3 | 42 | P3Q42i | Doze off: | 00  01  02  03  04  99 | Never  Less than once/week  1-2 times/week  3-4 times/week  5+ times/week  Don’t know/remember | 2 |
| Part 3 | 43 | P3Q43 | Hours of sleep for highest function | Hours  97 | Actual hours noted  Depends/varies | 2 |
| Part 3 | 44 | P3Q44a | Daily time spent watching TV | 00  01  02  03  99 | No time  Few minutes/less than 1 hr  1-3 hours  3+ hours  Don’t know/remember | 2 |
| Part 3 | 44 | P3Q44b | Daily time spent computer/Internet | 00  01  02  03  99 | No time  Few minutes/less than 1 hr  1-3 hours  3+ hours  Don’t know/remember | 2 |
| Part 3 | 44 | P3Q44c | Daily time spent reading | 00  01  02  03  99 | No time  Few minutes/less than 1 hr  1-3 hours  3+ hours  Don’t know/remember | 2 |
| Part 3 | 44 | P3Q44d | Daily time spent exercising | 00  01  02  03  99 | No time  Few minutes/less than 1 hr  1-3 hours  3+ hours  Don’t know/remember | 2 |
| Part 3 | 44 | P3Q44e | Daily time spent phone | 00  01  02  03  99 | No time  Few minutes/less than 1 hr  1-3 hours  3+ hours  Don’t know/remember | 2 |
| Part 3 | 44 | P3Q44f | Daily time spent socializing | 00  01  02  03  99 | No time  Few minutes/less than 1 hr  1-3 hours  3+ hours  Don’t know/remember | 2 |
| Part 3 | 44 | P3Q44g | Daily time spent cooking/eating | 00  01  02  03  99 | No time  Few minutes/less than 1 hr  1-3 hours  3+ hours  Don’t know/remember | 2 |
| Part 3 | 44 | P3Q44h | Daily time spent w/ friends/family | 00  01  02  03  99 | No time  Few minutes/less than 1 hr  1-3 hours  3+ hours  Don’t know/remember | 2 |
| Part 3 | 44 | P3Q44i | Daily time spent: (WRITE IN) | 00  01  02  03  99 | No time  Few minutes/less than 1 hr  1-3 hours  3+ hours  Don’t know/remember | 2 |
| Part 3 | 44 | P3Q44j | Daily time spent: (WRITE IN) | 00  01  02  03  99 | No time  Few minutes/less than 1 hr  1-3 hours  3+ hours  Don’t know/remember | 2 |
| Part 3 | 45 | P3Q45 | Enough time off between shifts | 00  01  99 | No  Yes  Not sure | 2 |
| Part 3 | 46 | P3Q46 | If workshift was shorter-would get more sleep | 00  01  99 | No  Yes  Not sure | 2 |
| Part 3 | 47 | P3Q47 | Time needed to wind down | 00  Minutes  Hours  97  99 | No time  Actual minutes noted  Actual hours noted  Depends/varies  Not sure | 2 |
| Part 3 | 48 | P3Q48 | Work days: time it takes to fall asleep | Minutes  Hours  97  99 | Actual minutes noted  Actual hours noted  Depends/varies  Don’t know/don’t remember | 2 |
| Part 3 | 49 | P3Q49 | Nonwork days: time it takes to fall asleep | Minutes  Hours  97  98  99 | Actual minutes noted  Actual hours noted  Depends/varies  Does not apply  Don’t know/don’t remember | 2 |
| Part 3 | 50 | P3Q50a | Over past 2 weeks, experienced: difficulty falling asleep | 00  01  02  03  04  99 | Never  Rarely  Sometimes  Frequently  Always  Don’t know/remember | 2 |
| Part 3 | 50 | P3Q50b | Over past 2 weeks, experienced: waking up during sleep | 00  01  02  03  04  99 | Never  Rarely  Sometimes  Frequently  Always  Don’t know/remember | 2 |
| Part 3 | 50 | P3Q50c | Over past 2 weeks, experienced: waking up too early/ being unable to fall back asleep | 00  01  02  03  04  99 | Never  Rarely  Sometimes  Frequently  Always  Don’t know/remember | 2 |
| Part 3 | 50 | P3Q50d | Over past 2 weeks, experienced: waking up feeling tired | 00  01  02  03  04  99 | Never  Rarely  Sometimes  Frequently  Always  Don’t know/remember | 2 |
| Part 3 | 50 | P3Q50e | Over past 2 weeks, experienced: sleeping soundly | 00  01  02  03  04  99 | Never  Rarely  Sometimes  Frequently  Always  Don’t know/remember | 2 |
| Part 3 | 50 | P3Q50f | Over past 2 weeks, experienced: sleeping through alarm | 00  01  02  03  04  99 | Never  Rarely  Sometimes  Frequently  Always  Don’t know/remember | 2 |
| Part 3 | 50 | P3Q50g | Over past 2 weeks, experienced: hitting snooze button | 00  01  02  03  04  99 | Never  Rarely  Sometimes  Frequently  Always  Don’t know/remember | 2 |
| Part 3 | 50 | P3Q50h | Over past 2 weeks, experienced: waking up from bad dreams | 00  01  02  03  04  99 | Never  Rarely  Sometimes  Frequently  Always  Don’t know/remember | 2 |
| Part 3 | 51 | P3Q51 | How long stays awake after waking in middle of night | Minutes  Hours  98 | Actual number of minutes  Actual number of hours  Does not apply | 2 |
| Part 3 | 52 | P3Q52 | Awakened during statutory rest period | 00  01  02  03  04 | Never  Rarely  Sometimes  Frequently  Always | 2 |
| Part 3 | 53 | P3Q53 | How long it takes to return to sleep | Minutes  Hours  98  99 | Actual number of minutes  Actual number of hours  Does not apply  Don’t know/remember | 2 |
| **PART IV – SLEEP PROBLEMS** | | | | | | |
| Part 4 | 54 | P4Q54 | Rest breaks | Minutes  Hours  98  99 | Actual number of minutes  Actual number of hours  Doesn’t apply/no breaks  Don’t know/remember | 2 |
| Part 4 | 55 | P4Q55 | Time allowed to deliver load | 00  01  02  03  04  98  99 | Never realistic  Rarely realistic  Sometimes realistic  Frequently realistic  Always realistic  Doesn’t apply/no dispatcher  Don’t know/haven’t thought about it | 2 |
| Part 4 | 56 | P4Q56 | Work over 14 hours/day | 00  01  02  03  04  99 | Never  Rarely  Sometimes  Frequently  Always  Don’t know/remember | 2 |
| Part 4 | 57 | P4Q57 | Work over weekly hour limit | 00  01  02  03  04  99 | Never  Rarely  Sometimes  Frequently  Always  Don’t know/remember | 2 |
| Part 4 | 58 | P4Q58 | Take fewer than 10 hours rest | 00  01  02  03  04  99 | Never  Rarely  Sometimes  Frequently  Always  Don’t know/remember | 2 |
| Part 4 | 59 | P4Q59 | Underreport work hours in logbook | 00  01  02  03  04  99 | Never  Rarely  Sometimes  Frequently  Always  Don’t know/remember | 2 |
| Part 4 | 60 | P4Q60 | Heard of drivers having more than 1 logbook | 00  01  99 | No  Yes  Don’t know/remember | 2 |
| Part 4 | 61 | P4Q61 | How often drove sleepy a vehicle other than truck in last month | 00  Number  98  99 | Never  Actual number of times  Does not apply  Don’t know/remember | 2 |
| Part 4 | 62 | P4Q62 | Drove truck while sleepy in last month | 00  Number  99 | Never  Actual number of times  Don’t know/remember | 2 |
| Part 4 | 63 | P4Q63 | Sleepiness impacted job performance | 00  01  02  03  04  05  99 | Never  Less than once/week  2-3 times/week  3-4 times/week  4-5 times/week  5+ times/week  Don’t know/remember | 2 |
| Part 4 | 64 | P4Q64 | Work days missed in last month due to sleepiness | 00  Number  99 | None  Actual number of times  Don’t know/remember | 2 |
| Part 4 | 65 | P4Q65 | Sleepiness impacted concentration | 00  01  02  03  04  05  99 | Never  Less than once/week  2-3 times/week  3-4 times/week  4-5 times/week  5+ times/week  Don’t know/remember | 2 |
| Part 4 | 66 | P4Q66a | Due to sleepiness: made serious error | 01  02  99 | Yes  No  Don’t know/remember | 2 |
| Part 4 | 66 | P4Q66b | Due to sleepiness: caused an accident | 01  02  99 | Yes  No  Don’t know/remember | 2 |
| Part 4 | 66 | P4Q66c | Due to sleepiness: in accident caused by another | 01  02  99 | Yes  No  Don’t know/remember | 2 |
| Part 4 | 66 | P4Q66d | Due to sleepiness: had near miss | 01  02  99 | Yes  No  Don’t know/remember | 2 |
| Part 4 | 66 | P4Q66e | Due to sleepiness: had crash | 01  02  99 | Yes  No  Don’t know/remember | 2 |
| Part 4 | 66 | P4Q66f | Due to sleepiness: got injured | 01  02  99 | Yes  No  Don’t know/remember | 2 |
| Part 4 | 66 | P4Q66g | Due to sleepiness: injured others | 01  02  99 | Yes  No  Don’t know/remember | 2 |
| Part 4 | 66 | P4Q66h | Due to sleepiness: had injury requiring medical attention | 01  02  99 | Yes  No  Don’t know/remember | 2 |
| Part 4 | 67 | P4Q67a | Impact of insufficient sleep on work | 00  01  02  98  99 | No impact (WATCH ORDER OF ANSWERS)  Some impact  Major impact  Not applicable  Don’t know/remember | 2 |
| Part 4 | 67 | P4Q67b | Impact of insufficient sleep on social life/leisure activities | 00  01  02  98  99 | No impact (WATCH ORDER OF ANSWERS)  Some impact  Major impact  Not applicable  Don’t know/remember | 2 |
| Part 4 | 67 | P4Q67c | Impact of insufficient sleep on family life/home responsibilities | 00  01  02  98  99 | No impact (WATCH ORDER OF ANSWERS)  Some impact  Major impact  Not applicable  Don’t know/remember | 2 |
| Part 4 | 67 | P4Q67d | Impact of insufficient sleep on mood | 00  01  02  98  99 | No impact (WATCH ORDER OF ANSWERS)  Some impact  Major impact  Not applicable  Don’t know/remember | 2 |
| Part 4 | 67 | P4Q67e | Impact of insufficient sleep on intimate/sexual relations | 00  01  02  98  99 | No impact (WATCH ORDER OF ANSWERS)  Some impact  Major impact  Not applicable  Don’t know/remember | 2 |
| Part 4 | 67 | P4Q67f | Impact of insufficient sleep on physical health | 00  01  02  98  99 | No impact (WATCH ORDER OF ANSWERS)  Some impact  Major impact  Not applicable  Don’t know/remember | 2 |
| Part 4 | 67 | P4Q67g | Impact of insufficient sleep on mental health | 00  01  02  98  99 | No impact (WATCH ORDER OF ANSWERS)  Some impact  Major impact  Not applicable  Don’t know/remember | 2 |
| Part 4 | 68 | P4Q68a | Walk in sleep | 00  01  02  03  04  99 | Never  Rarely  Sometimes  Frequently  Always  Don’t know/remember | 2 |
| Part 4 | 68 | P4Q68b | Talk in sleep | 00  01  02  03  04  99 | Never  Rarely  Sometimes  Frequently  Always  Don’t know/remember | 2 |
| Part 4 | 68 | P4Q68c | Kick legs in sleep | 00  01  02  03  04  99 | Never  Rarely  Sometimes  Frequently  Always  Don’t know/remember | 2 |
| Part 4 | 68 | P4Q68d | Grind teeth/clench jaw in sleep | 00  01  02  03  04  99 | Never  Rarely  Sometimes  Frequently  Always  Don’t know/remember | 2 |
| Part 4 | 68 | P4Q68e | Gasp, choke, snort in sleep | 00  01  02  03  04  99 | Never  Rarely  Sometimes  Frequently  Always  Don’t know/remember | 2 |
| Part 4 | 68 | P4Q68f | Stop breathing in sleep | 00  01  02  03  04  99 | Never  Rarely  Sometimes  Frequently  Always  Don’t know/remember | 2 |
| Part 4 | 68 | P4Q68g | Have frightening dreams in sleep | 00  01  02  03  04  99 | Never  Rarely  Sometimes  Frequently  Always  Don’t know/remember | 2 |
| Part 4 | 68 | P4Q68h | Have leg cramps in sleep | 00  01  02  03  04  99 | Never  Rarely  Sometimes  Frequently  Always  Don’t know/remember | 2 |
| Part 4 | 68 | P4Q68i | Snore loudly in sleep | 00  01  02  03  04  99 | Never  Rarely  Sometimes  Frequently  Always  Don’t know/remember | 2 |
| Part 4 | 68 | P4Q68j | Other | 00  01  02  03  04  99 | Never  Rarely  Sometimes  Frequently  Always  Don’t know/remember | 2 |
|  |  |  |  | 05 | String variable (write out) | 10 |
| Part 4 | 69 | P4Q69a | Diagnosed with sleep apnea | 00  01  99 | No (WATCH ORDER OF ANSWERS)  Yes  Don’t know/remember | 2 |
| Part 4 | 69 | P4Q69b | Diagnosed with shiftwork sleep disorder | 00  01  99 | No (WATCH ORDER OF ANSWERS)  Yes  Don’t know/remember | 2 |
| Part 4 | 69 | P4Q69c | Diagnosed with insomnia | 00  01  99 | No (WATCH ORDER OF ANSWERS)  Yes  Don’t know/remember | 2 |
| Part 4 | 69 | P4Q69d | Diagnosed with sleep hypopnea | 00  01  99 | No (WATCH ORDER OF ANSWERS)  Yes  Don’t know/remember | 2 |
| Part 4 | 69 | P4Q69e | Diagnosed with restless leg syndrome | 00  01  99 | No (WATCH ORDER OF ANSWERS)  Yes  Don’t know/remember | 2 |
| Part 4 | 69 | P4Q69f | Other | 00  01  99 | No (WATCH ORDER OF ANSWERS)  Yes  Don’t know/remember | 2 |
|  |  |  |  | 03 | String variable (write out) | 10 |
| Part 4 | 70 | P4Q70 | Prescribed medication for sleep disorder | 00  01  99 | No  Yes  Don’t know/remember | 2 |
| Part 4 | 71 | P4Q71a | Adavan (or Ativan) *(Lorazepam)* | 00  01  02  03  99 | Never used  Have used in the past  Currently use  Prescribed/never use  Don’t know/remember | 2 |
| Part 4 | 71 | P4Q71b | Ambien *(Zolpidem Tartrate)* | 00  01  02  03  99 | Never used  Have used in the past  Currently use  Prescribed/never use  Don’t know/remember | 2 |
| Part 4 | 71 | P4Q71c | Anafranil *(Chlomipramine)* | 00  01  02  03  99 | Never used  Have used in the past  Currently use  Prescribed/never use  Don’t know/remember | 2 |
| Part 4 | 71 | P4Q71d | Celexa *(Citalopram)* | 00  01  02  03  99 | Never used  Have used in the past  Currently use  Prescribed/never use  Don’t know/remember | 2 |
| Part 4 | 71 | P4Q71e | Desyrel *(Desipramine)* | 00  01  02  03  99 | Never used  Have used in the past  Currently use  Prescribed/never use  Don’t know/remember | 2 |
| Part 4 | 71 | P4Q71f | Effexor *(Venlafaxine)* | 00  01  02  03  99 | Never used  Have used in the past  Currently use  Prescribed/never use  Don’t know/remember | 2 |
| Part 4 | 71 | P4Q71g | Lexapro *(Escitalopram)* | 00  01  02  03  99 | Never used  Have used in the past  Currently use  Prescribed/never use  Don’t know/remember | 2 |
| Part 4 | 71 | P4Q71h | Lunesta *(Eszopiclone)* | 00  01  02  03  99 | Never used  Have used in the past  Currently use  Prescribed/never use  Don’t know/remember | 2 |
| Part 4 | 71 | P4Q71i | Luvox *(Fluvoxamine)* | 00  01  02  03  99 | Never used  Have used in the past  Currently use  Prescribed/never use  Don’t know/remember | 2 |
| Part 4 | 71 | P4Q71j | Melatonin | 00  01  02  03  99 | Never used  Have used in the past  Currently use  Prescribed/never use  Don’t know/remember | 2 |
| Part 4 | 71 | P4Q71k | Mirapex *(Pramipexole)* | 00  01  02  03  99 | Never used  Have used in the past  Currently use  Prescribed/never use  Don’t know/remember | 2 |
| Part 4 | 71 | P4Q71l | Neurontin *(Gabapentin)* | 00  01  02  03  99 | Never used  Have used in the past  Currently use  Prescribed/never use  Don’t know/remember | 2 |
| Part 4 | 71 | P4Q71m | Paxil *(Paroxetine)* | 00  01  02  03  99 | Never used  Have used in the past  Currently use  Prescribed/never use  Don’t know/remember | 2 |
| Part 4 | 71 | P4Q71n | Provigil *(Modafinil)* | 00  01  02  03  99 | Never used  Have used in the past  Currently use  Prescribed/never use  Don’t know/remember | 2 |
| Part 4 | 71 | P4Q71o | Prozac *(Fluoxetine)* | 00  01  02  03  99 | Never used  Have used in the past  Currently use  Prescribed/never use  Don’t know/remember | 2 |
| Part 4 | 71 | P4Q71p | Remeron *(Mirtazapine)* | 00  01  02  03  99 | Never used  Have used in the past  Currently use  Prescribed/never use  Don’t know/remember | 2 |
| Part 4 | 71 | P4Q71q | Requip *(Ropinirole)* | 00  01  02  03  99 | Never used  Have used in the past  Currently use  Prescribed/never use  Don’t know/remember | 2 |
| Part 4 | 71 | P4Q71r | Restoril *(Temazepam)* | 00  01  02  03  99 | Never used  Have used in the past  Currently use  Prescribed/never use  Don’t know/remember | 2 |
| Part 4 | 71 | P4Q71s | Ritalin *(Methylphenidate)* | 00  01  02  03  99 | Never used  Have used in the past  Currently use  Prescribed/never use  Don’t know/remember | 2 |
| Part 4 | 71 | P4Q71t | Rozerem *(Ramelteon)* | 00  01  02  03  99 | Never used  Have used in the past  Currently use  Prescribed/never use  Don’t know/remember | 2 |
| Part 4 | 71 | P4Q71u | Serzone *(Nefazodone)* | 00  01  02  03  99 | Never used  Have used in the past  Currently use  Prescribed/never use  Don’t know/remember | 2 |
| Part 4 | 71 | P4Q71v | Silenor *(Doxepin)* | 00  01  02  03  99 | Never used  Have used in the past  Currently use  Prescribed/never use  Don’t know/remember | 2 |
| Part 4 | 71 | P4Q71w | Sinemet *(Carbidopa Levotopa)* | 00  01  02  03  99 | Never used  Have used in the past  Currently use  Prescribed/never use  Don’t know/remember | 2 |
| Part 4 | 71 | P4Q71x | Sonata *(Zaleplon)* | 00  01  02  03  99 | Never used  Have used in the past  Currently use  Prescribed/never use  Don’t know/remember | 2 |
| Part 4 | 71 | P4Q71y | Strattera *(Atomoxetine)* | 00  01  02  03  99 | Never used  Have used in the past  Currently use  Prescribed/never use  Don’t know/remember | 2 |
| Part 4 | 71 | P4Q71z | Tofranil *(Imipramine)* | 00  01  02  03  99 | Never used  Have used in the past  Currently use  Prescribed/never use  Don’t know/remember | 2 |
| Part 4 | 71 | P4Q71aa | Xanax *(Alprazolam)* | 00  01  02  03  99 | Never used  Have used in the past  Currently use  Prescribed/never use  Don’t know/remember | 2 |
| Part 4 | 71 | P4Q71ab | Zoloft *(Sertraline)* | 00  01  02  03  99 | Never used  Have used in the past  Currently use  Prescribed/never use  Don’t know/remember | 2 |
| Part 4 | 72 | P4Q72 | CPAP therapy use | 00  01  98  99 | No  Yes  Doesn’t apply/no sleep apnea  Don’t know/not sure | 2 |
| AlPart 4 | 73 | P4Q73 | Other substances used to sleep in past year | 00  01  02  03  04  05  06  07  99 | Never used  Once  2-6 times/past year  7-12 times/past year  1-4 times/month  5-8 times/month  3-5 times/week  Daily  Don’t know/remember | 2 |
|  |  |  |  | 97 | Participant wanted to skip question | 2 |
| **PART V - BACKGROUND** | | | | | | |
| Part 5 | 74 | P5Q74 | Age | Number | Write actual age in years | 2 |
| Part 5 | 75 | P5Q75 | Race/ethnicity | 01  02  03  04  05  06  07  08 | White/Caucasian  Black/African American  Latino/Hispanic  White Hispanic  Asian  Native American/Am Indian  Multiracial/multiethnic  Other | 2 |
|  |  |  |  | 09 | String variable (write out) | 10 |
| Part 5 | 76 | P5Q76 | Education | 01  02  03  04  05  06  07  08 | Some high school  High school diploma/GED  Some trade school  Trade school  Some college  College degree  Some grad/professnl school  Grad/professional degree | 2 |
| Part 5 | 77 | P5Q77 | Last year’s personal income (after taxes) | 01  02  03  04  05  06  07  08 | $10,000-$20,000  $20,000-$30,000  $30,000-$40,000  $40,000-$50,000  $50,000-$60,000  $60,000-$70,000  $70,000-$80,000  $80,000 or more | 2 |
| Part 5 | 78 | P5Q78 | Compensation type | 01  02  03  04 | By the mile  By the load  Percentage of revenue  Other | 2 |
|  |  |  |  | 05 | String variable (write out) | 10 |
| Part 5 | 79 | P5Q79 | Healthcare coverage | 00  01  02  03 | No insurance  Private insurance  Government insurance  Other | 2 |
|  |  |  |  | 04 | String variable (write out) | 10 |
| Part 5 | 80 | P5Q80 | Who pays for healthcare insurance | 01  02  03  04  05  99 | Driver alone pays/out-of-pocket  Driver and employer  Spouse’s employer  Government  Other  Don’t know/remember/unsure | 2 |
|  |  |  |  | 06 | String variable (write out) | 10 |
| Part 5 | 81 | P5Q81 | Driver pays for others/his employees | 01  02  03  99 | Health insurance  Workman’s compensation  Other  Does not apply | 2 |
|  |  |  |  | 04 | String variable (write out) | 10 |
| Part 5 | 82 | P5Q82 | Union membership | 00  01 | No  Yes | 2 |
|  |  |  |  | 03 | String variable (write out union or association name) | 10 |
| **PART VI – FUELING PREFERENCES (FOR TA)** | | | | | | |
| Part 6 | 83 | P6Q83 | Fleet dictates which truckstop to fuel at | 00  01  02  03  04  98 | Never  Rarely  Sometimes  Frequently  Always  Does not apply | 2 |
| Part 6 | 84 | P6Q84 | Truckstops told to fuel at | 01  02  03  98 | Pumper-only truckstops  Full-service truckstops  Other independent truckstop  Does not apply | 2 |
| Part 6 | 85 | P6Q85 | Driver preference of truckstops | 01  02  03 | Pumper-only stops  Full-service truckstops  Other independent truckstop | 2 |
| Part 6 | 86 | P6Q86 | Preference of last 5 fueling stops at brands other than the TA | 00  01  02  03  04  98 | Never  Rarely  Sometimes  Frequently  Always  Does not apply | 2 |
| Part 6 | 87 | P6Q87 | Likelihood of changing fleets for more truckstop fueling choices | 01  02  03  04  05  98 | Not at all likely  Slightly likely  Moderately likely  Very likely  Completely likely  Does not apply | 2 |
